# Assessment of content validity for a Neonatal Near miss Scale in the context of Ethiopia

**DOI:** 10.1080/16549716.2021.1983121

**Published:** 2021-10-25

**Authors:** Mengstu Melkamu Asaye, Kassahun Alemu Gelaye, Yohannes Hailu Matebe, Helena Lindgren, Kerstin Erlandsson

**Affiliations:** a Department of Women and Family Health, School of Midwifery, College of Medicine and Health Sciences, University of Gondar, Gondar, Ethiopia; b Department of Epidemiology and Biostatistics, Institute of Public Health, College of Medicine and Health Sciences, University of Gondar, Gondar, Ethiopia; c Department of Pediatrics and Child Health, School of Medicine, College of Medicine and Health Sciences, University of Gondar, Gondar, Ethiopia; d Department of Women’s and Children’s Health, KarolinskaInstitute, Solna, Sweden; e Department of Women’s and Children’s Health, KarolinskaInstitute and Institution for Health and Welfare, Dalarna University, Solna, Sweden

**Keywords:** Domain, expert panel, scale, neonatal near-miss, validation

## Abstract

**Background:**

The concept of neonatal near miss is used to identify neonates who nearly died but survived a life-threatening complication in the first 28 days of life. Neonatal mortality is the tip of the iceberg. Quality improvement through utilization of a validated scale and reduction in adverse neonatal outcome is a priority for achieving sustainable development goals.

**Objectives:**

To develop and assess the content validity of neonatal near-miss scale in the public health hospitals in Amhara Regional State, northwest Ethiopia.

**Methods:**

A literature review was performed prior to the development of the neonatal near-miss assessment scale. An expert panel committee was formed by health facility practitioners and by the members of the academia. Two rounds of meetings were conducted with the expert panel to reach consensus on the face and content validity. The content validity index, Kappa statistics, and the content validity ratio were computed to estimate the content validity scale of neonatal near miss.

**Results:**

In this study, four domains (pragmatic, clinical, management, and lab-investigations) with 32 items were identified. The item-level content validity index ranged from 0.7 to 1. The overall scale content validity (S-CVI) (average) for the domains (pragmatic, clinical, management, and lab-investigations) were 0.98, 0.95, 0.96, and 0.96, respectively. The overall S-CVI (universal) was 0.78 to 1, whereas the overall S-CVI (average) of neonatal near miss assessment scale was found to be 0.96. The content validity ratio and Kappa statistics values ranged from 0.6 to 1 and 0.9 to 1 for the respective domains.

**Conclusion:**

The identified four domains and the respective items were valid enough (content-wise) to be used as identification criteria for neonatal near-miss cases. The scale will contribute to neonatal near-miss identification and also improve the quality of neonatal management care.

## Background

In 2017, 2.5 million neonates died globally in the first month of life, representing an average of about 7,000 every day. Most of these deaths occurred in the first week of birth. Based on this, consequently 28 million newborns are estimated to die between 2018 and 2030, and 80% of these deaths would occur in Southern Asia and sub-Saharan Africa [1].

However, Ethiopia had outlined a plan to reduce the neonatal mortality rate (NMR from 29 per 1,000 live births in 2015/2016 to 11 per 1,000 live births by 2019/2020 [2], but increased to 30 per 1,000 live births in 2019 [3]. The highest mortality was in Amhara Regional State, which had an NMR of about 47 per 1,000 live births [4].

Neonatal mortality is a significant public health problem in many low-resource countries [5]. However, for every death, there are more than eight newborns that suffer life-threatening complications but survive (near-miss) [6]. The concept of neonatal near-miss is a recent term and used to explain neonates who nearly died but survived from life-threatening complications during the first 28 days of extra-uterine life. It is becoming an increasingly important indicator not only for epidemiologic surveillance but also for assessment of quality of care [7].

Neonatal mortality is the tip of the iceberg, but we also see a higher number of ill survivors than the number of deaths due to a lack of a validated assessment scale [8]. According to a multi-country study carried out by the WHO, ideally, one near-miss case would mirror one death.The only difference could be that the neonate was alive at the time of assessing the vital status [9]. Neonatal near-miss data should be used together with neonatal mortality data as a tool in the assessment of quality of care provision [10,11].

According to a data-based analysis of WHO cross-sectional studies, the concept of neonatal near miss and scale development was useful for shaping improvements in health care and of the health systems towards achieving Millennium Development Goals 4 and 5 [9].

Several scoring tools have been used to assess severe neonatal morbidities, but none of these scoring markers can be used to define near miss neonates [12]. The emerging pragmatic criteria are birth weight under 1,750 g, an APGAR score under 7 after 5 minutes, and gestational age under 33 completed gestation weeks [9,13–17]. The management criteria are phototherapy within 24 hours of life, cardiopulmonary resuscitation, use of vasoactive drugs, anticonvulsants, blood product or surfactant utilization, surgery, or use of steroids for treatment of refractory hypoglycemia, or intubation for 7 days [18–21]. One study used certain clinical criteria [22]. Lab-investigation criteria were not included, but they could be feasible in low-resource countries like Ethiopia. The validated neonatal near-miss assessment scale could be simple to use and easily understandable [9].

Development and validation of neonatal near miss criteria could facilitate the use of a neonatal near miss scale as the measurement of quality of neonatal care and for the evaluation of death reviews [8,10]. Unlike maternal near miss, currently, there is no standard definition of a near miss neonate or a content and face validated neonatal near-miss assessment scale [6,9,13,23]. This makes the development and content validity a challenge before scaling up such activities [6,8,10,12,24,25].

Focusing on near-miss cases allows identification of a sufficient number of cases to study and understanding of health system failures within a short-time period, as compared to neonatal death studies. On top of this, studying neonatal near miss to identify health system failures is more acceptable for health care providers, as it would be a good opportunity to give feedback [23].

Evidence suggest that researching neonatal near-miss cases rather than only neonatal deaths can provide more information on what goes wrong as the sample is larger, the parents are more available to give feedback, and the obstetric and neonatal staff can improve their practice by avoiding blaming each other [12,23,26,27].

The conceptualization and operationalization of a neonatal near-miss scale in the local context of Ethiopia need further information on interventions and performances useful for shaping improvements in neonatal health care and the health systems, with the goal of achieving Sustainable Development Goals [9].

There is limited evidence in Ethiopia describing the process of developing a context-specific neonatal near-miss scale based on face and content validity for large-scale use in Ethiopian neonatal wards [23]. This study aimed to develop and validate, content wise, the context-specific neonatal near-miss assessment scale and was conducted at the University of Gondar, College of Medicine and Health Science, and the University of Gondar Comprehensive Specialized Hospital in Amhara Regional State, northwest Ethiopia.

## Methods

### Design

In this study, two steps from theoretical background/literature review and experts’ opinions to develop and content validate the neonatal near-miss assessment scale were used. Theoretical background/literature review was performed prior to the development of the neonatal near miss assessment scale. Then, an expert panel committee was formed from members of the academia (pediatrics and neonatal health, clinical midwifery, reproductive health, epidemiology, and biostatistics) and health facility practitioners (neonatal nurses and midwives). Experts participating in this study were informed that their participation was entirely voluntary, and they were free to withdraw at any time. Two rounds of meetings were conducted with the expert panel with the aim of reaching consensus on the face and content validity.

### Assessment scale development steps

The absence of a validated identification scale for near-miss cases makes it very difficult to establish the relationship between near-miss cases and neonatal deaths. Contextual validated scale development could allow comparisons between different settings, regardless of local development level and across time [28]. The initial steps of scale development were performed using a three-step approach: identifying the content domain, generating the sample items, and constructing the scale [29].

### Domain identification

The content domain of the construct of neonatal near-miss is identified through literature review, content analysis, and expert panel discussions [30]. The literature review helped the researchers identify different research gaps in the foundation of the near-miss neonates and their assessments instrument [7]. Consensus-based standards for selection of health status measurements instrument (COSMIN) checklist was also used [31]. During this preliminary work, the conceptualization of the central concept of items under each domain was emphasized. Pragmatic and management domains were selected from a previous study [25] but with the addition and deletion of more than seven items, certain clinical [22] and lab investigation scales were added from the literature [32] and experts’ suggestion based on their feasibility order in the low resource setting study area. Finally, four domains with 31 items were approved for the identification of near miss neonates’ cases (pragmatic, management, clinical, and laboratory criteria).

### Item generations

Items in each domain were presented hierarchically with easier and more feasible items at the top of each domain and less feasible ones at the bottom [33]. The items developed for neonatal near-miss identification were reviewed by an expert panel committee. The panels of experts were selected considering expert knowledge, specific training, or professional experience on the subject matter (Table 1).

**Table 1.** Sociodemographic data of the expert panel on phase one scale from the public health hospital of Amhara Regional State, northwest Ethiopia, 2020

Panel	Designation	Expertise	Organization	Experience
1	Ph.D. (Asso.professor)	Public health	IPH,University of Gondar (UOG)	>20 years
2	MD(Asst.professor)	Pediatrician	School of Medicine,UOG	>9 years
3	MSc(Lecturer)	Pediatrics and child health nursing	School of Nursing,UOG	>12 years
4	Asso.professor	Clinical midwifery and epidemiology	School of Midwifery,UOG	>17 years
5	BSc(Practitioner)	Neonatal nurse	Neonatology ward,UOG	>7 years
6	MSc(Practitioner)	Clinical midwifery	Maternity ward,UOG	>8 years
7	Asst.professor	Ph.D. student and clinical midwifery	School of Midwifery,UOG	>11 years
8	Asst.professor	Ph.D. student and clinical midwifery	School of Midwifery,UOG	>10 years
9	Asst.professor	Pediatrics and child health nursing	School of Nursing,UOG	13 years

### Content validity

**Figure 1.**
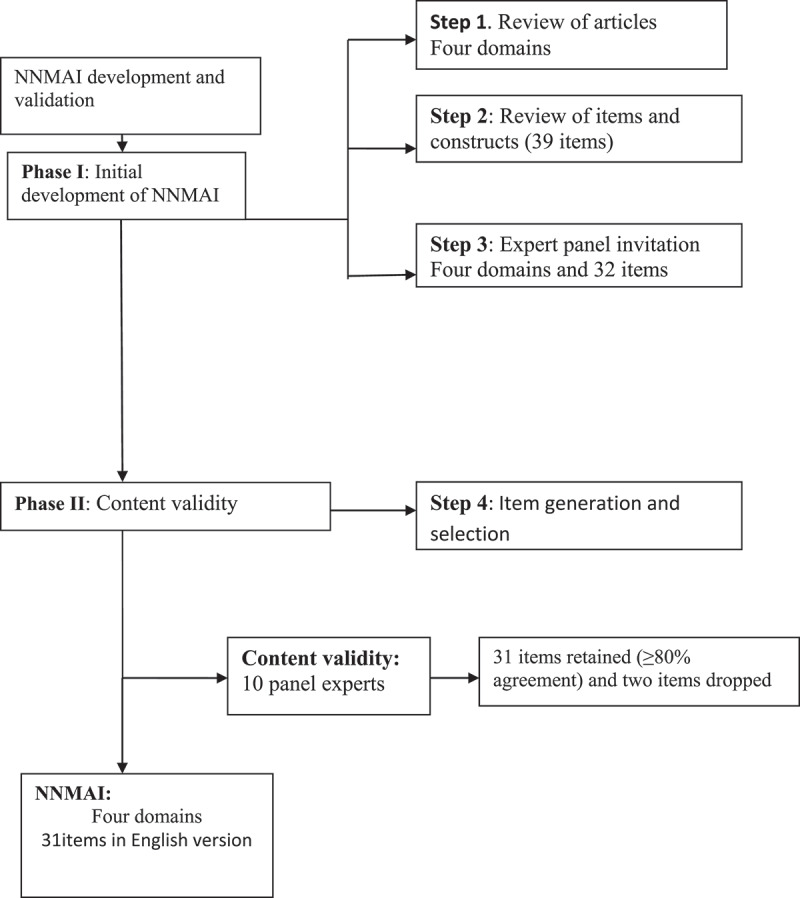
Steps for development and assessment of content validity for a neonatal near miss scale in the context of Ethiopia

Content validity is a precondition for other forms of statistical validity. It assesses the dimensions of the construct intended to be measured and reflects a specific domain of content. It helps the researchers gain invaluable feedback from panel experts [30]. Addressing content validity begins with scale development. An invitation letter was sent via email to nine expert panel members with detailed explanations and the neonatal near-miss assessment scale one week before the first panel meeting. Then, after the expert panels had given their judgments individually, we contacted them through phone call to schedule a face-to-face meeting. The meeting took 2,5 hours. The panel meeting aimed to approve/add/delete the identified four domains by literature review, and to evaluate the items in each domain, as well as to ensure their relevance to assess the construct and neonatal near miss. The items with domains feasibility, representativeness, and applicability in low-resource setting hospitals were also assessed by the panel members during the panel meeting. All the experts who attended the panel meeting had reached a consensus on approving a total of four domains and 32 items with comments (seven items were eliminated, two items relocated to other domains, and order rearrangement was done). After this, a reviewed version was resubmitted to experts for approval through email with either all comments that were raised during the meeting incorporated or not. We received approval from all panel experts. Then, we designed a preliminary version of NNMAS comprising of 32 items grouped into four domains (Figure 1).

To minimize over or under estimation for the quantifications, 10 other independent panels of experts were invited for the second round to assess the necessity, relevancy, and clarity of each selected item in measuring the related domains. This panel was selected based on their expert knowledge in the field, specific training, and professional experience on the subject matter, with consideration of work experience of five or more years (Table 2).

**Table 2.** Sociodemographic data of the expert panel on phase two scale in the public health hospital of Amhara Regional State, northwestern Ethiopia, 2020

Panel	Designation	Expertise	Organization	Experience
1	PhD(Asst.professor)	RH and epidemiologist	IPH,University of Gondar (UOG)	>12 years
2	MD(Asst.professor)	Pediatrician	School of Medicine,UOG	>5 years
3	MD(Asst.professor)	Pediatrician	School of Medicine,UOG	>6 years
4	Asst.professor	Pediatrics and child health	School of Nursing,UOG	>12 years
5	BSc(practitioner)	Senior midwife	Maternity ward,UOG,hospital	>7 years
6	BSc(practitioner)	Neonatal nurse	Neonatology ward,UOG	>5 years
7	Asst.professor	Clinical midwifery	School of Midwifery,UOG	>10 years
8	Asst.professor	Clinical midwifery	School of Midwifery,UOG	>11 years
9	Asst.professor	Epidemiologist and Ph.D. student	Institute of Public Health,UOG	>10 years
10	Asst.professor	Clinical midwifery and Ph.D. student	School of Midwifery,UOG	>16 years

This expert panel were also asked to give their professional judgment on the scoring rate by considering the representativeness of individual items, whether the items in each domain adequately measured what they intended to measure and were asked to suggest revisions, additions and/or deletion of items in each construct. They also gave a score for each item based on the completeness, feasibility and time used for application, which was 20 minutes for all items, in each domain. The quantitative viewpoints on the relevance, necessity, clarity, and representativeness were collected to ensure the content validity of the items generated.

### Quantification of content validity

#### Content validity ratio

According to the Lawshe test [34], content validity ratio (CVR) was computed to specify whether an item is necessary for operating a construct or not. The experts were asked to give a score of (1 = not essential, 2 = useful but not essential, and 3 = essential.)

CVR = (Ne – N/2)/(N/2)

Ne-stands for the number of panelists indicating ‘essential’ and N is the total number of panelists. CVR values range between −1(perfect disagreement) and +1(perfect agreement) with CVR values above zero, indicating that over half of panel members agree on an item being essential [35].

#### Content validity index (CVI)

The CVI was calculated for all individual items (I-CVI) and the overall scale (S-CVI). Experts were asked to rate each scale item in terms of its relevance to the underlying construct. The four points used along the item rating continuum were 1 = not relevant, 2 = somewhat relevant, 3 = quite relevant, and 4 = highly relevant.

### Individual items

#### Content validity index (I-CVI) = (3or4)/N

The number of experts giving a rating of (3 or 4 = 1); N = total number of experts who were involved, and I-CVI was not less than 0.78.

##### Scale-content validity index (S-CVI)

This can be conceptualized in two ways: S-CVI (universal agreement) and S-CVI (average). The S-CVI (universal agreement) reflects the proportion of items on the scale that achieved a rating of 3 or 4 by all the experts on the panel. This shows the experts’ performance level. S-CVI (average) emphasizes average item quality rather than the average performance of the experts. It is recommended that an S-CVI should be 0.8 at a minimum for reflecting content validity [30].

##### Kappa statistics coefficient

CVI is extensively used by researchers. However, it does not take into consideration the inflated values that may occur because of the possibility of chance agreement. Thus, computation of the Kappa statistics coefficient ensures a better understanding of content validity, as it removes any random chance agreement. Kappa statistic is a consensus index of inter-rater agreement that supplements CVI to ensure that the agreement among experts is beyond chance. Computation of Kappa statistics require the calculation of the probability of change agreement, that is, Pc = [N/A (N – A)]× 0.5 ^N^, where N = number of experts in the panel, A = number of experts in the panel who agree that the item is relevant. Kappa statistics are then calculated as K = (I-CVI – Pc)/(1 – Pc). Values above 0.74, between 0.60 and 0.74, and between 0.40 and 0.59 are considered to be excellent, good, and fair, respectively [30].

## Results

In the current study, a panel of 19 experts was involved in two rounds. It comprised the members of the academia (experts in pediatrics and neonatal health, clinical midwifery, reproductive health, epidemiology, and biostatistics) and health facility practitioners (neonatal nurses and midwives) with more than 5 years of work experience.

Under the essentiality of items quantified by the experts, more than 98% of the items’ content validity ratio was in the range of 0.60 to 1.00. This indicates that the items are necessary (content valid) in order to assess neonatal near-miss cases. In this study, CVR of hematuria was found to be 0.4, which is below 0.5 and therefore considered not necessary (Table 3). The I-CVI for all the items in the four domains ranged from 0.70 to 1.00. The S-CVI (average) for pragmatic, management, clinical, and lab-investigation domains of NNMAS was found to be 0.97, 0.95, 0.96, and 0.96, respectively (Table 3).

**Table 3.** CVR for items from each domain with a number of experts who gave a scoring rate for an item in the public health hospital of Amhara Regional State, northwest Ethiopia, 2020

Domains	Items	Expert1	Expert2	Expert3	Expert 4	Expert5	Expert6	Expert 7	Expert8	Expert9	Expert10	Ne	CVR
**Pragmatic**	APGAR score<7 at 5 m	**3**	**3**	**3**	**3**	**3**	**3**	**3**	**1**	**3**	**3**	**9**	**0.8**
	Birth weight <1,750 g	**3**	**3**	**3**	**3**	**3**	**3**	**3**	**3**	**3**	**3**	**10**	**1**
	Gestational age <34 weeks	**3**	**3**	**3**	**3**	**3**	**3**	**3**	**2**	**3**	**3**	**9**	**0.8**
	Auxiliary temperature (12–24 hours)	**3**	**3**	**3**	**3**	**3**	**3**	**3**	**3**	**3**	**3**	**10**	**1**
**Clinical**	Absence of regular breathing	**3**	**3**	**3**	**3**	**3**	**3**	**3**	**2**	**3**	**3**	**9**	**0.8**
	Cardiac arrest(asystole)	**2**	**3**	**3**	**3**	**3**	**3**	**3**	**3**	**3**	**3**	**9**	**0.8**
	Respiratory rate >70 bpm	**3**	**3**	**3**	**3**	**3**	**3**	**3**	**3**	**3**	**3**	**10**	**1**
	Bradycardia<80 bpm	**3**	**3**	**3**	**3**	**3**	**3**	**3**	**3**	**3**	**3**	**10**	**1**
	Jaundice duringfirst 24 hours	**3**	**3**	**3**	**3**	**3**	**3**	**3**	**3**	**3**	**3**	**10**	**1**
	Tachycardia >180 bpm	**2**	**3**	**3**	**3**	**3**	**3**	**3**	**3**	**3**	**3**	**9**	**0.8**
	Recurrent seizures(three or more episodes)	**2**	**3**	**3**	**3**	**3**	**3**	**3**	**3**	**3**	**3**	**9**	**0.8**
	Inability to suck within 12 hours	**1**	**3**	**3**	**3**	**3**	**3**	**3**	**3**	**3**	**3**	**9**	**0.8**
	Nural tube defects	**2**	**3**	**3**	**3**	**3**	**3**	**2**	**3**	**3**	**3**	**8**	**0.6**
	Haematuria	**1**	**3**	**3**	**3**	**3**	**3**	**2**	**3**	**3**	**2**	**7**	0.4
	Persistent central cyanosis	**3**	**3**	**3**	**3**	**3**	**3**	**3**	**2**	**3**	**3**	**9**	**0.8**
	Non passage of urine >24 hours	**2**	**3**	**3**	**3**	**3**	**3**	**3**	**3**	**3**	**3**	**9**	**0.8**
	Vomiting(threeand more episodes)	**3**	**3**	**3**	**3**	**3**	**3**	**3**	**3**	**3**	**3**	**10**	**1**
**Management**	Nasal CPAP	**2**	**3**	**3**	**3**	**3**	**3**	**3**	**3**	**3**	**3**	**9**	**0.8**
	Phototherapy within 24 hours	**3**	**3**	**3**	**3**	**3**	**3**	**3**	**3**	**3**	**3**	**10**	**1**
	Chest compression	**3**	**3**	**3**	**3**	**3**	**3**	**3**	**3**	**3**	**3**	**10**	**1**
	Positive pressure ventilation	**3**	**3**	**3**	**3**	**3**	**3**	**3**	**3**	**3**	**2**	**9**	**0.8**
	Use of any vasoactive drugs	**2**	**3**	**3**	**3**	**3**	**3**	**3**	**3**	**3**	**3**	**9**	**0.8**
	Use of anticonvulsants	**2**	**3**	**3**	**3**	**3**	**3**	**3**	**2**	**3**	**3**	**8**	**0.8**
	Blood exchange transfusion	**2**	**3**	**3**	**3**	**3**	**3**	**3**	**3**	**3**	**3**	**9**	**0.8**
	Use of corticosteroid for hypoglycemia	**3**	**3**	**3**	**3**	**3**	**3**	**3**	**3**	**3**	**3**	**9**	**1**
	Major surgery in the first week	**3**	**3**	**3**	**3**	**3**	**3**	**3**	**2**	**3**	**3**	**9**	**0.8**
	Intubation for suctioning	**3**	**3**	**3**	**3**	**3**	**3**	**3**	**3**	**3**	**3**	**10**	**1**
**Lab-investigation**	Hgb<10 g/dl	**3**	**3**	**3**	**3**	**3**	**3**	**3**	**2**	**3**	**3**	**9**	**0.8**
	WBC <4,000cells/mm^3^	**3**	**3**	**3**	**3**	**3**	**3**	**3**	**3**	**3**	**3**	**10**	**1**
	Serum blirubinelevel>10 mg/dl within 24 hours	**3**	**3**	**3**	**3**	**3**	**3**	**3**	**2**	**3**	**3**	**9**	**0.8**
	Was blood culture positive?	**3**	**3**	**3**	**3**	**3**	**3**	**3**	**3**	**3**	**3**	**10**	**1**
	Blood glucose level <40 mg/dl in 24 hours	**3**	**3**	**3**	**3**	**3**	**3**	**3**	**3**	**3**	**3**	**10**	**1**

*Note:Ne-number of panel experts indicated essential and **CVR** (Content Validity Ratio).

The overall S-CVI (universal) for the 32-items scale ranged between 0.78 and 1.00, which indicated the high content validity of the items for the construct of the neonatal near miss assessment scale. The overall S-CVI (average) of NNMAS was found to be 0.96 (Table 3). After quantification, we have produced the final version of the NNMAS scale containing 31 items under four domains. One item (hematuria), with 70% agreement was rejected (Table 4).

**Table 4.** Ratings on NNMAS by 10 experts’ items rated 3 or 4 on a 4-point relevant scale in the public health hospital of Amhara Regional State, northwestern Ethiopia, 2020

Domains	Item	Expert1	Expert2	Expert3	Expert4	Expert5	Expert6	Expert 7	Expert8	Expert9	Expert10	No. In Agreement	I-CVI	PC	Kappa Statistics
**Pragmatic**	**1**	**4**	**4**	**3**	**4**	**4**	**4**	**4**	**2**	**4**	**4**	**9**	**0.9**	**0.00976**	**0.899**
	**2**	**3**	**4**	**4**	**4**	**4**	**4**	**4**	**3**	**4**	**4**	**10**	**1**	**0.000976**	**1**
	**3**	**4**	**4**	**4**	**4**	**4**	**4**	**4**	**4**	**4**	**4**	**10**	**1**	**0.000976**	**1**
	**4**	**4**	**4**	**4**	**3**	**4**	**4**	**4**	**3**	**4**	**4**	**10**	**1**	**0.000976**	**1**
**Clinical**	**5**	**4**	**4**	**4**	**3**	**4**	**4**	**4**	**3**	**4**	**4**	**10**	**1**	**0.000976**	**1**
	**6**	**3**	**4**	**4**	**3**	**4**	**4**	**4**	**4**	**4**	**4**	**10**	**1**	**0.000976**	**1**
	**7**	**4**	**4**	**4**	**4**	**4**	**4**	**3**	**3**	**3**	**4**	**10**	**1**	**0.000976**	**1**
	**8**	**4**	**4**	**4**	**4**	**4**	**4**	**4**	**4**	**4**	**4**	**10**	**1**	**0.000976**	**1**
	**9**	**4**	**4**	**4**	**4**	**4**	**4**	**4**	**3**	**3**	**4**	**10**	**1**	**0.000976**	**1**
	**10**	**2**	**4**	**4**	**4**	**4**	**4**	**3**	**4**	**4**	**4**	**9**	**0.9**	**0.00976**	**0.899**
	**11**	**3**	**4**	**4**	**4**	**4**	**4**	**3**	**3**	**3**	**4**	**10**	**1**	**0.000976**	**1**
	**12**	**1**	**4**	**4**	**4**	**4**	**4**	**4**	**3**	**4**	**4**	**9**	**0.9**	**0.00976**	**0.899**
	**13**	**3**	**4**	**4**	**4**	**4**	**4**	**3**	**4**	**3**	**4**	**10**	**1**	**0.000976**	**1**
	**14**	**1**	**4**	**4**	**4**	**4**	**2**	**3**	**4**	**4**	**2**	**7**	**0.7**	**0.703125**	**Negative**
	**15**	**3**	**4**	**4**	**4**	**4**	**4**	**4**	**3**	**4**	**4**	**10**	**1**	**0.000976**	**1**
	**16**	**4**	**1**	**3**	**4**	**4**	**4**	**3**	**4**	**3**	**3**	**9**	**0.9**	**0.00976**	**0.899**
	**17**	**4**	**4**	**4**	**4**	**4**	**4**	**4**	**4**	**4**	**4**	**10**	**1**	**0.000976**	**1**
**Management**	**18**	**4**	**4**	**3**	**4**	**4**	**3**	**4**	**3**	**4**	**2**	**9**	**0.9**	**0.00976**	**0.899**
	**19**	**2**	**4**	**4**	**4**	**4**	**4**	**4**	**4**	**4**	**4**	**9**	**0.9**	**0.00976**	**0.899**
	**20**	**1**	**4**	**4**	**4**	**4**	**4**	**4**	**3**	**4**	**4**	**9**	**0.9**	**0.00976**	**0.899**
	**21**	**2**	**4**	**4**	**4**	**4**	**3**	**4**	**4**	**4**	**4**	**9**	**0.9**	**0.00976**	**0.899**
	**22**	**3**	**4**	**4**	**4**	**4**	**4**	**4**	**3**	**4**	**4**	**10**	**1**	**0.000976**	**1**
	**23**	**4**	**4**	**4**	**4**	**4**	**4**	**4**	**3**	**3**	**4**	**10**	**1**	**0.000976**	**1**
	**24**	**4**	**4**	**4**	**4**	**4**	**4**	**4**	**4**	**4**	**4**	**10**	**1**	**0.000976**	**1**
	**25**	**4**	**4**	**4**	**4**	**4**	**4**	**4**	**4**	**4**	**4**	**10**	**1**	**0.000976**	**1**
	**26**	**4**	**4**	**4**	**4**	**4**	**4**	**3**	**3**	**4**	**4**	**10**	**1**	**0.000976**	**1**
	**27**	**3**	**4**	**3**	**4**	**4**	**4**	**3**	**4**	**4**	**3**	**10**	**1**	**0.000976**	**1**
**Labinvestigation**	**28**	**2**	**4**	**4**	**4**	**4**	**4**	**4**	**4**	**4**	**3**	**9**	**0.9**	**0.000976**	**0.899**
	**29**	**4**	**4**	**3**	**4**	**4**	**4**	**4**	**4**	**4**	**4**	**10**	**1**	**0.000976**	**1**
	**30**	**4**	**4**	**4**	**4**	**4**	**4**	**4**	**3**	**4**	**4**	**10**	**1**	**0.000976**	**1**
	**31**	**4**	**4**	**4**	**4**	**4**	**4**	**4**	**4**	**1**	**4**	**9**	**0.9**	**0.00976**	**0.899**
	**32**	**4**	**4**	**4**	**4**	**4**	**4**	**4**	**3**	**4**	**3**	**10**	**1**	**0.000976**	**1**
**S-CVI(un)**		**0.78**	**0.97**	**1**	**1**	**1**	**0.97**	**1**	**0.97**	.**97**	**1**	**S-CVI(ave) = 0.96**			

**Note**: S-CVI (un) = scale level content validity index (universal) and S-CVI (ave) = scale content validity index (average).

## Discussion

This study aimed to describe the development and content validity of a context-specific neonatal near miss assessment scale for use in an Ethiopian low-resource setting. Many researchers have used and described various neonatal near-miss tools, and some have been validated [9,25]. No researchers have examined the impact of validated and reliable neonatal near miss tools, and the authors filled this specific gap in this paper. We have taken the first step in providing a contextually valid version of NNMAS that could provide valid, representative, and easily administered criteria for neonatal near-miss cases in low-resource settings in countries like Ethiopia. This can save the lives of neonates and reduce the high burden of neonatal death [8].

Universal access to quality neonatal health services is essential to meet specific sustainable development goals to reduce neonatal and overall child mortality. Data for decision-making are crucial for planning services and monitoring progress [36]. A neonatal near miss scale can be used as a measure of the quality of neonatal care and to evaluate death reviews [10]. Quality of care could be measured using these standards. Thus, it could help to improve the quality of care in the clinical practice [37].

Based on the experts’ suggestions, certain changes in the wording and rearrangement of the order of items and clarifications were made. Except for minor wording modifications, the experts on the panel for face validity did not provide suggestions regarding item deletion or addition. The criteria developed and validated were simple to use, highly related with near miss and death, and could be served as diagnosis and predictor of later mortality [11,20]. The scale needs to be accepted and used by health care providers in neonatal wards. Being familiar with a scale emphasizes the importance of face-to-face introduction. Furthermore, the invitation to health care providers to be involved at the neonatal ward is critical [13]. The usefulness of this tool (scale) was proven in this study of face and content validation of the neonatal near miss assessment scale in this local context and could answer many researchers’ questions, although it must be further validated [6,8–10,23,25]. This study added clinical and simple lab-investigation domains with nearly 20 items that could be implemented in low-resource countries. These results were supported by the face and content validity, which was a qualitative measure required as an important first step in the development of the scale [33]. In the current study, the overall S-CVI (universal) for the 31-item scale ranged between 0.78 and 1.00, and the overall S-CVI (average) of NNMAS was found to be 0.96. This indicated the high content validity of the items for the construction of the neonatal near miss assessment scale.

### Limitation of the study

After confirmation of face and content validity, other types of validity and reliability need to be considered in the NNMAS validation process. The main limitation is the relatively advanced quantitative measures on a relatively small sample, despite us having invited 10 other panel experts (six academics, two practitioners, and two methodologists) to avoid unnecessary, potential biases.

The validation process of NNMAS therefore still needs other types of validity and its prospective predictive capability needs further evaluation for full implementation. To produce valid results, the content of a test, survey, or measurement method must cover all relevant parts of the subject. It aims to measure [30]. If some aspects are missing from the measurement, or if irrelevant aspects are included, the validity is threatened [38]. We therefore suggest psychometric testing to provide a solid foundation for tool validation.

## Conclusion and clinical implications

Face and content validity are the first developmental phase for full psychometrical validation of NNMAS as this is a unique scale, and all the quantification findings indicated that this validated and reliable tool could be implemented in low-resource countries, to identify neonatal near-miss cases and, potentially, as such, support health care providers with a tool that will support decision-making, which in turn will help reduce the neonatal near miss morbidity and mortality in low-resource settings, not only in Ethiopia. The NNMAS showed face validity with minor rewording following suggestions from experts and holds a promise to identify near-miss neonates. Testing the validity and reliability of the scale with full psychometric properties and testing its comprehensiveness for respondents could be extremely important.

## List of abbreviations

APGAR= Appearance, Pulse, Grimace, Activity, and Respiration

COSMIN=Consensus-based Standards for the selection of health status Measurement Instrument; CVI=Content Validity Index; CVR=Content Validity Ratio; I-CVI=Item-Content Validity Index; NNMAS=Neonatal Near miss Assessment Scales; S-CVI=Scale-Content Validity Index; UOG=University of Gondar; WHO=World Health Organization

## Paper context

In Ethiopia, the neonatal death estimate is only the tip of the iceberg. The concept of neonatal near miss is used to identify why neonates who nearly died survived from a life-threatening complication during the first 28 days of life. This paper adds a validated near miss assessment scale for the Ethiopian context to the topic. This study provides a validated scale that can be used to identify neonatal near-miss cases in Ethiopia.
